# Rainfall-Associated Bronchospasm Epidemics: The Epidemiological Effects of Air Pollutants and Weather Variables

**DOI:** 10.1155/2017/9252069

**Published:** 2017-09-27

**Authors:** Kambiz Masoumi, Maryam Haddadzadeh Shoushtari, Arash Forouzan, Ali Asgari Darian, Maryam Dastoorpoor, Pegah Ebrahimzadeh, Hamidreza Aghababaeian

**Affiliations:** ^1^Department of Emergency Medicine, Imam Khomeini General Hospital, Ahvaz Jundishapur University of Medical Sciences, Ahvaz, Iran; ^2^Air Pollution and Respiratory Diseases Research Center, Ahvaz Jundishapur University of Medical Sciences, Ahvaz, Iran; ^3^Department of Epidemiology and Biostatistics, Faculty of Public Health, Ahvaz Jundishapur University of Medical Sciences, Ahvaz, Iran; ^4^Nursing and Emergency Department, Dezful University of Medical Sciences, Dezful, Iran

## Abstract

**Background:**

This study compares different risk factors in patients visiting a hospital during five rainfall-associated bronchospasm epidemics in Ahvaz and those visiting on other occasions.

**Methods:**

This case-control study was conducted on 5307 patients with bronchospasm admitted to the Emergency Department of Imam Khomeini Hospital in Ahvaz (Iran) from late October to December (as the epidemic) and 916 patients admitted from late January to March (as the nonepidemic) in 2011 to 2015.

**Results:**

A total of the 41.7% of the cases and 48.8% of the controls had episodes of bronchospasm, suggesting a significant difference between the two groups (*P* < 0.001). The mean concentrations of PM_10_, NO, NO_2_, and NO_*x*_ pollutants (except O_3_) were significantly higher in the nonepidemic periods (*P* < 0.05). The adjusted analysis showed a direct significant relationship between emergency respiratory admissions and each unit of increase in NO and SO_2_ concentration during the epidemic periods and NO_2_ concentration during the nonepidemic periods. During the epidemic periods, a direct and significant relationship was also observed between respiratory admissions and each unit of increase in relative humidity and evaporation.

**Conclusion:**

The results suggest that certain pollutants and weather variables are associated with the risk of emergency respiratory admissions during epidemic periods.

## 1. Introduction

Allergic diseases such as asthma and bronchospasm are considered a major global health problem that costs billions of dollars to health systems in the world for treatment and compensation for the disabilities associated with them [1, 2]. Asthma and bronchospasm are a multifactorial diseases of the airways that develop due to both a very strong genetic predisposition and climatic changes and contact with allergens [3]. Climatic changes have a dramatic effect on the incidence of asthma and bronchospasm attacks. Evidence also suggests that asthma epidemic is much more common in the pollen season and during thunderstorms and heavy rainfalls [4, 5].

Several mechanisms are involved in the incidence of these diseases, with the most well-known being the exacerbation of viral infection, increased concentrations of stimulating gases in the air (such as ozone), and changes in the concentrations of allergens and microparticles of dust [6–8]. The flow of humid air causes osmotic pressure in the plant pollen and paves the way for their disintegration, thereby creating microparticles that can be absorbed through the airways. This process occurs mostly in the morning and following the first rainfall of the year. Moreover, thunderstorms and lightening create an electrical charge in the allergens present in the air and facilitate the dispersion and release of microparticles [6, 9]. In 2014, the International Panel on Climate Change reported that the rise in greenhouse gases such as nitrogen dioxide and carbon dioxide can affect temperature changes and lead to more severe climatic phenomena such as storms, snowfalls, heavy rainfalls, hurricanes, tornados, and big hails [5]. Many studies have also shown that contact with pollutants such as sulfur dioxide, ozone, nitrogen dioxide, and particulates smaller than 10 microns can cause or exacerbate allergic and respiratory diseases such as bronchial asthma and allergic rhinitis [10–13].

Other studies have shown that air pollution increases allergenicity and the accumulation of allergenic pollens in the environment. In addition, the pollen of plants that grow in regions with high air pollution has a higher allergenicity. Higher allergenicity can increase allergies and exacerbate allergic responses to the allergens present in the respiratory tract of patients with bronchospasm [14–16]. Asthma and bronchospasm attacks due to rainfall and thunderstorm have been a major problem for the health system in the past two decades. Heavy rainfalls and thunderstorms increase the referral and hospitalization of patients with asthma and bronchospasm and thus impose heavy costs on the society [3].

In the past two decades, many epidemics have been reported after heavy rainfalls [5]. The main risk factors identified as the cause of these epidemics include rising temperatures, increased rainfall and thunderstorm, and increased plant allergens following rainfall [8]. These epidemics have been reported in different parts of the world; for example, in 1983, this epidemic led to a fivefold increase in emergency hospital visits in Birmingham, UK, and similar epidemics were reported in Melbourne, Australia, in 1987 and 1989 [3]. An asthma epidemic following a thunderstorm and rainfall on June 24, 1994, in London led to 640 emergency hospital visits for asthma and other respiratory problems in only 30 minutes, which was ten times higher than the expected rate of referral. More than half of the patients were 21 to 40 years old and 403 (about 63%) had a history of seasonal asthma and allergies while 382 (about 42%) were experiencing asthma attacks for the first time [17].

Ahvaz, a city in Khouzestan Province of Iran, has witnessed a dramatic increase in the incidence of asthma and bronchospasm attacks following heavy rainfalls; a new phenomenon has emerged in this city every year from 2011 following the first autumn rainfall, which has been given the title “the Respiratory Crisis” due to its continuous emergence. Since 2011, following the first autumn rainfall, hospitals in this city have admitted an overwhelming number of patients presenting with respiratory symptoms, especially shortness of breath and coughs, and this sudden and unusual rush of patients has continued to increase, so that, by sunrise the next morning of a rainfall, ten hours after the rainfall, at most, 20,000 emergency hospital visits were made for respiratory symptoms in 2013, 10,400 visits in 2014, and 26,400 visits in 2015 [18].

The level of air pollution in Ahvaz increases and becomes more severe day by day. According to a 2011 World Health Organization report, Ahvaz had an average annual particulate concentration of 372 *µ*g/m^3^ for particulates smaller than 10 microns (PM_10_) and was thus identified as the most polluted city in the world [19]. Given the large number of emergency hospital admissions for respiratory problems and the substantially heavy costs of their treatment, this study was conducted to compare clinical, epidemiological, and environmental (air pollutants and weather variables) risk factors in patients visiting a select hospital of Ahvaz during thunderstorm-associated asthma epidemics and those visiting on other occasions.

## 2. Methods

This case-control study was designed first to compare clinical and epidemiological risk factors in 7–60-year-old patients presenting with respiratory symptoms including shortness of breath, wheezing, coughing, and phlegm to the Emergency Department of Imam Khomeini Hospital during the epidemic periods of late October to December and those presenting during the nonepidemic periods of late January to March in 2011 to 2015. Those presenting during the rainfall- or thunderstorm-associated asthma or bronchospasm epidemic period (60 days) over the five years were considered the case group and those presenting during the nonepidemic period (60 days) were taken as the control group. Some of the patients had visited the hospital more than once; however, they were included in their group as a single subject. The age range studied was seven to 60 so as to be able to exclude patients with COPD (older than 60) [20] and children with virus-related wheezing (younger than seven) [21].

The hospital studied was a fully equipped public teaching, medical, and research referral center in Ahvaz with a Division of Pulmonary and Respiratory Diseases that admits people from different parts of the city (covering an area of 528 km^2^) and from different socioeconomic backgrounds.

The data collection tool was a three-part researcher-designed questionnaire. Part one inquired about the patients' sociodemographic information, including age, gender, and history of smoking. Part two dealt with their clinical data, including symptoms upon admission (wheezing, shortness of breath, coughing, and phlegm), history of asthma and allergic diseases, the recurrence of symptoms in later years, the duration of stay in the emergency department, and the progression of the disease in the emergency department (discharge/hospitalization). Part three inquired about the patients' medical data, including the type of treatment received in the emergency department, the need for oxygenation, and history of pharmacotherapy for respiratory problems. The questionnaires were completed for both the epidemic and nonepidemic periods based on the emergency admission records of the hospital. The researchers contacted the patients over the phone to complete their demographic details and assess the rates of their disease recurrence in later years.

This ecological study was also designed to compare pollutant concentrations and weather variables in the epidemic and nonepidemic periods of the disease over the given five-year period.

Data was obtained on seven pollutants registered at Khouzestan Province Environmental Protection Agency, including ozone (O_3_), sulfur dioxide (SO_2_), nitrogen dioxide (NO_2_), nitrogen oxides (NO_*x*_), carbon monoxide (CO), and particulates less than 10 microns in diameter (PM_10_), and the 24-hour values of these pollutants were extracted as their mean daily values. The mean, minimum, and maximum temperature, the mean relative humidity, the amount of precipitation, total evaporation, and wind speed and direction were obtained on a daily basis from Khuzestan Province Meteorological Office.

**Figure 1:**
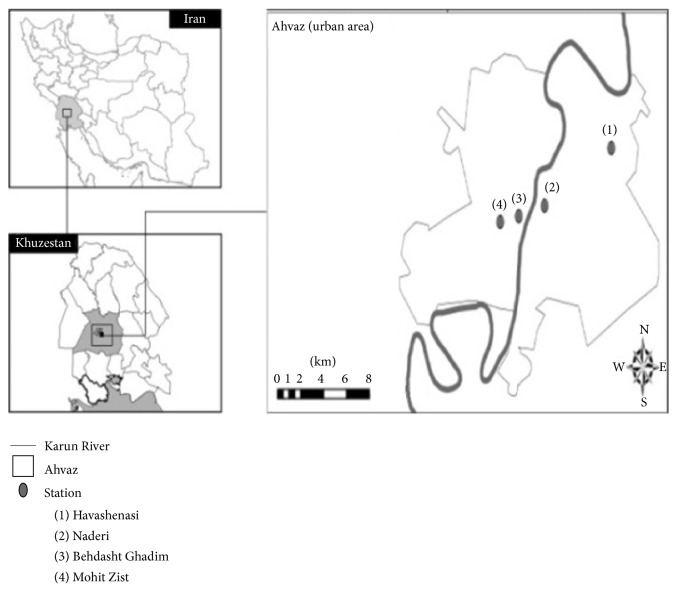
Air quality monitoring stations in Ahvaz.

There are four air quality monitoring stations in Ahvaz, including the Environmental Protection Agency station, the Naderi Square station, the University Square station, and the Meteorological Office station, and the mean daily values extracted from the four stations were used as the reference value in this study. According to experts at the Environmental Protection Agency in Ahvaz, air quality monitoring stations are located as such in this city because they are a good representation of the air pollution index for the entire city (Figure 1).

The data obtained from other studies shows that the concentration of pollutants in Ahvaz changes by the hour, day, week, and month, but not by location [19]. It can therefore be argued that people who visit the hospital from different parts of the city are exposed to almost the same concentrations of pollutants.

With an area of 528 km^2^, Ahvaz is the capital of Khuzestan Province and is located in Southeast Iran at the northern latitude of 31 degrees and 32 minutes and the eastern longitude of 48 degrees and 68 minutes [19]. According to a 2011 census, Ahvaz is home to 286.032 households and has a population of 1.11 million [22].

Ethical issues (plagiarism, informed consent, research misconduct, data fabrication and/or falsification, double publication and/or submission, redundancy, etc.) have been completely observed by the authors. The Ethics Committee of Ahvaz Jundishapur University of Medical Sciences approved the study protocol. Informed consent (oral and written) of all participants was obtained and regulations of the Declaration of Helsinki were followed throughout the study.

A descriptive analysis was used to assess the descriptive variables of the study, including the number of hospital visits for respiratory problems during the epidemic and nonepidemic periods, the concentration of pollutants, and weather variables. The *X*^2^-test was used to assess the relationship between the qualitative variables, and the odds ratio was calculated to determine the risk factors. The *t*-test was also used to compare pollutant concentrations in the epidemic and nonepidemic periods.

A negative binomial regression was finally used to assess the relationship between the number of emergency respiratory admissions, the mean daily concentration of pollutants, and weather variables separately by the type of period assessed, that is, epidemic and nonepidemic. The analysis was performed in STATA-11 at a significance level of less than 0.05.

## 3. Results

From 2011 to 2015, 5307 people visited the emergency department of the select hospital with respiratory complaints (shortness of breath, wheezing, coughing, and phlegm) from late October to December and made up the case group of thunderstorm or rainfall-associated asthma or bronchospasm, and 916 people visited from late January to March and made up the control group of nonepidemic hospital visits for respiratory problems.

**Figure 2:**
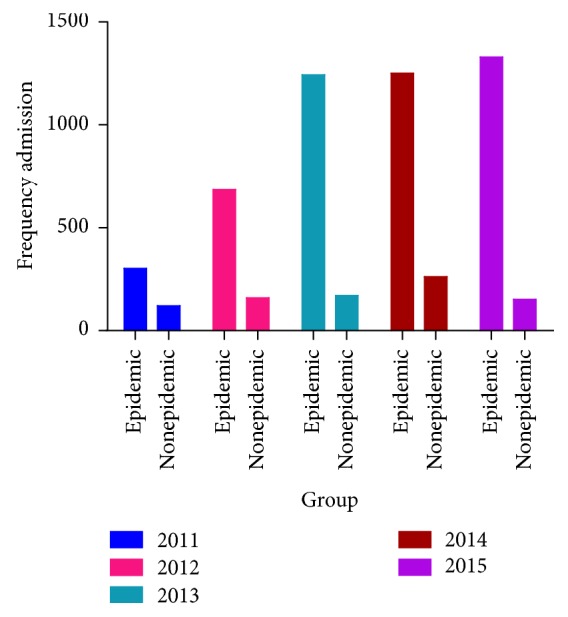
Frequency distribution of number of admissions during the epidemic and nonepidemic periods in the five years of study.

The lowest rate of hospital admission during the asthma or bronchospasm epidemic period was observed in 2011 (302 people) and the highest in 2015 (1329 people), which indicates the ascending trend of hospital admission for respiratory problems during the epidemic periods of these five years (Figure 2).

**Figure 3:**
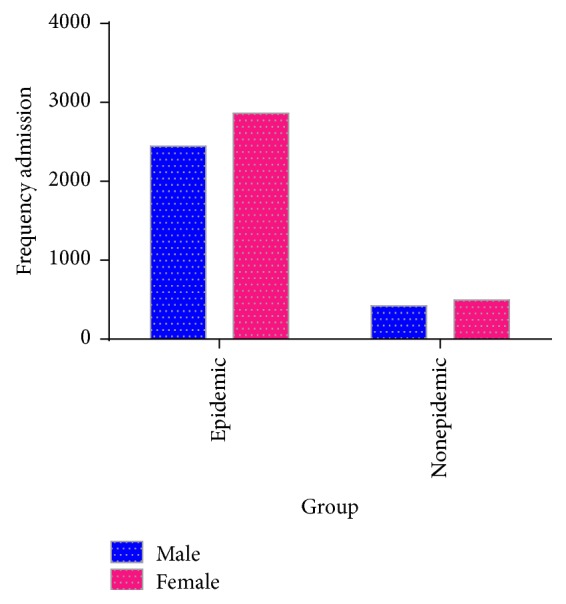
Frequency distribution of number of admissions during the epidemic and nonepidemic periods according to gender.

The majority of the admitted patients were female in both the case (53.9%, *n* = 2862) and the control (54%, *n* = 495) groups (Figure 3).

**Figure 4:**
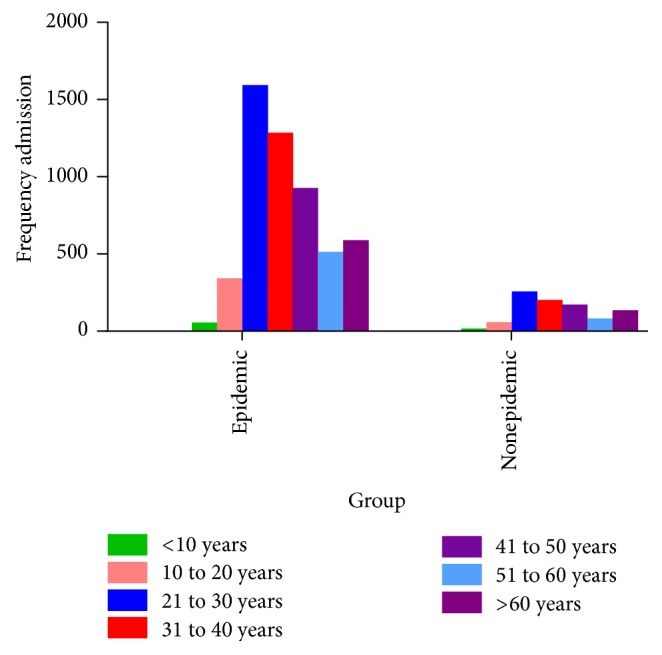
Frequency distribution of number of admissions during the epidemic and nonepidemic periods according to age groups.

In terms of age, the majority (30.04%) of the cases involved in the outbreak were in the 21–30 age group, followed by 22.53% in the 31–40 age group. The majority (28.17%) of the controls were also in the 21–30 age group (Figure 4).

The results obtained showed that 46.1% of the cases and 46% of the controls were male and the rest were female, which suggests the lack of significant differences between the two groups (*P* = 0.951).

In terms of age, 92.5% of the cases and 93% of the controls were aged 20 and older, which suggests the lack of significant differences between the two groups in terms of age (*P* = 0.585).

A total of 41.7% of the cases and 48.8% of the controls had a history of asthma or episodes of bronchospasm, which makes for a significant difference between the two groups (*P* < 0.001). In other words, the odds of having a history of asthma or bronchospasm attack were 25% lower in the cases than in the controls.

The prevalence of smoking was 64.5% among the cases and 57.6% among the controls, which makes for a significant intergroup difference; in other words, the odds of smoking were 1.3 times or 30% higher in the case group than in the controls.

The odds of hospitalization after emergency admission were 1.6 times higher in the case group than in the controls; in other words, people visiting emergency departments during rainfall or thunderstorm-associated asthma or bronchospasm epidemics were 40% more likely to be hospitalized compared to the controls.

No significant differences were observed between the two groups in terms of the prevalence of respiratory symptoms including wheezing, shortness of breath, and coughing, but the groups were significantly different in terms of the prevalence of phlegm, as the odds of having phlegm were 30% lower in the cases than in the controls (OR = 0.69).

No significant differences were observed between the two groups in terms of the history of allergic diseases, history of using medications for asthma, and the recurrence of the disease in later years.  Table 1 presents the other details pertaining to the cases and controls.

The patients were treated for their bronchospasm attacks using inhaled beta-agonist (spray/nebulizer), corticosteroid (inhaled, oral, or injectable), ipratropium bromide (spray/nebulizer), intravenous magnesium sulfate, subcutaneous epinephrine, and oxygenation depending on the clinical severity of their condition and regardless of whether they visited during the epidemic or the nonepidemic months.

As shown in Table 2, a significant difference was observed between the concentration of most air pollutants in the rainfall or thunderstorm-associated asthma or bronchospasm epidemic periods and in the nonepidemic periods, as the mean concentration of O_3_ was significantly higher in the epidemic (32.4) than in the nonepidemic (26.6) period (*P* = 0.001), while the mean concentrations of PM_10_, NO, NO_2_, and NO_*x*_ were significantly higher in the nonepidemic period.

The results obtained on weather variables showed that the mean relative humidity (58.86) and the amount of precipitation (35.33) were significantly higher in the epidemic period, while the evaporation rate was significantly lower in the epidemic period (*P* < 0.001).

Tables 3 and 4 present the relationship between respiratory hospital visits and the concentrations of pollutants for the entire population during both the epidemic and nonepidemic periods using the negative binomial regression.

The results of the crude analysis of the data for the epidemic periods showed a significant relationship between NO concentration and emergency respiratory visits; that is, the risk of emergency respiratory visits increased by 0.8% per each unit of increase in the mean daily concentration of NO, and this relationship remained significant even after adjusting the other confounding variables.

The results of the adjusted analysis of the data for the epidemic periods showed a significant relationship between SO_2_ concentration and emergency respiratory visits; that is, the risk of emergency respiratory visits increased by 1.4% per each unit of increase in the mean daily concentration of SO_2_.

The results of the crude analysis of the data for the nonepidemic periods showed a significant relationship between O_3_ concentration and respiratory hospital admissions; that is, respiratory admissions increased with each unit of increase in O_3_ concentration; however, this relationship was no longer significant after the adjustment of the confounding variables.

The results of the adjusted analysis for the nonepidemic periods showed a significant relationship between NO_2_ concentration and respiratory hospital admissions; that is, a 1% increase was observed in the risk of emergency respiratory admissions per each unit of increase in NO_2_ concentration.

The results of the crude analysis of the data for the epidemic periods showed a significant relationship between precipitation and respiratory hospital admissions; that is, each unit of increase in precipitation also increased respiratory admissions; however, this strong relationship was no longer significant after the adjustment of the confounding variables.

The results of the adjusted analysis for the epidemic periods showed a significant relationship between relative humidity and evaporation and the number of respiratory hospital admissions; that is, the risk of emergency respiratory admissions increased by 41.8% and 13% per each unit of increase in the mean daily values of relative humidity and evaporation.

**Figure 5:**
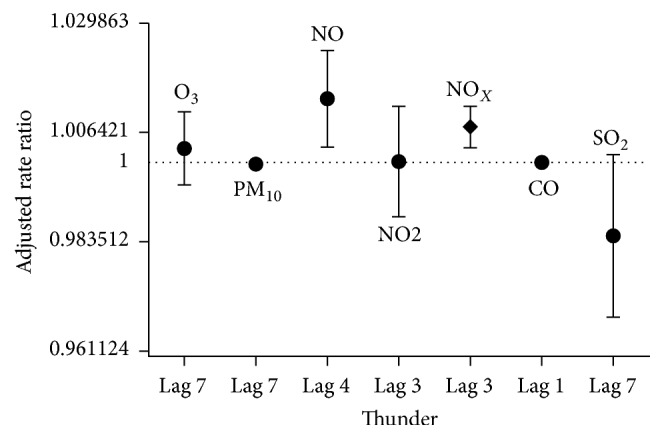
Strongest correlations between air pollutants and hospital admissions for asthma and bronchospasm happening up to 7 days later in epidemic periods.

The epidemic periods results of negative binomial adjusted-lag regression models with 0- to 7-day lags for exposure to pollutants showed a significant positive association between NO and NO_*x*_ and the number of respiratory hospital admissions at 4- and 3-day lags, respectively. No significant or positive relationships were observed between respiratory admissions and the concentrations of the other pollutants in the other negative binomial adjusted-lag regression models during the epidemic periods (Figure 5).

**Figure 6:**
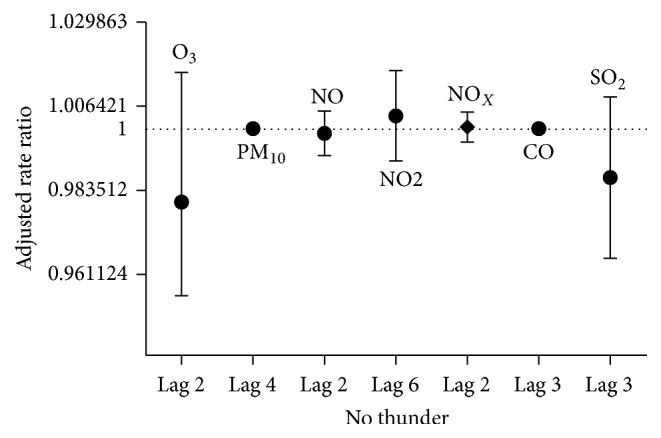
Strongest correlations between air pollutants and hospital admissions for asthma and bronchospasm happening up to 7 days later in nonepidemic periods.

The results of the adjusted-lag model of negative binomial regression for the nonepidemic periods to examine the zero- to seven-day lag following exposure to pollutants showed no significant relationships between the pollutant concentrations and respiratory hospital admissions (Figure 6).

## 4. Discussion

This study examined the data pertaining to five consecutive years and showed that rainfall and thunderstorm-associated asthma or bronchospasm epidemics occur every year in early autumn in Ahvaz. Rainfall and thunderstorm-associated asthma or bronchospasm epidemics were first identified around 1985 in the UK by Packe and Ayres, who showed that the number of hospital visits for rainfall and thunderstorm-associated asthma or bronchospasm is nine times larger than the number of visits for respiratory problems before these precipitations [23]. There are many reports on asthma epidemics following precipitation, including one report from London in the midnight of June 24 and 25, 1994 [17], another report in Wagga, Australia, on October 30, 1997 [24], and another one in Naples, Italy, on June 4, 2004 [25, 26].

The present study showed that the odds of being a smoker were 30% higher in the case group compared to the controls. This finding concurs with the results obtained by Ho et al. (2007), who showed that more people had a history of smoking in the group experiencing asthma attacks [27].

In the present study, less people had a history of asthma and bronchospasm in the case group compared to the controls, and no significant differences were observed between the two groups in terms of the use of asthma medications, allergic diseases, and the recurrence of the attacks in later years. In other words, most people who suffered during the epidemic period had no history of asthma or bronchospasm attacks. In one study, Girgis et al. (2000) argued that the risk of asthma or bronchospasm attacks during precipitation is higher in patients with a history of asthma and those who use certain asthma medications [24]. It can be concluded that the patients admitted during the nonepidemic periods mainly had pulmonary diseases including asthma or bronchospasm and were experiencing exacerbated attacks for various reasons. Moreover, although less people had a history of asthma or bronchospasm in the case group, the history of the use of asthma or bronchospasm medications was not different between the two groups; a possible conclusion can be that some of the patients with asthma or bronchospasm did not present to the clinic or hospital for treatment and follow-up before in epidemic group.

Overall, it can be argued that people with a history of smoking, asthma, bronchospasm, and asthma medication use should be careful about how often they leave home in the autumn and during the first autumn rainfalls.

The assessment of the concentration of the pollutants during the epidemic and nonepidemic periods showed that most pollutants (PM_10_, NO, NO_2_, and NO_*x*_) had a significantly lower concentration during the rainfall and thunderstorm-associated asthma or bronchospasm epidemic period compared to the nonepidemic period, and only O_3_ concentration was higher in the epidemic period, which suggests that a high concentration for most pollutants may not be the main cause of this epidemic.

These epidemics have also occurred in some other countries when the concentration of pollutants such as NO_2_ and PM_10_ have been well within the acceptable WHO standard range [3, 28]. Epidemiological evidence suggests a relationship between O_3_ concentration and emergency visits and hospitalizations for asthma. In one study [29], Wilson et al. (2005) showed that one (first quartile-third quartile) IQR increase in O_3_ concentration increases asthma-related emergency visits by 5% (95% CI: 1–10%). In another study [30], Lam et al. (2016) revealed the odds ratio of asthma-related hospitalization as 1.19 (95% CI: 1.07 to 1.32) for the single-pollutant model and as 1.44 (95% CI: 1.19 to 1.73) for the multipollutant model per each 10 *µ*g/m^3^ of increase in O_3_, which are considered statistically significant. In another study conducted in Milan, Italy [31], Santus et al. (2012) examined the relationship between emergency hospital admissions and environmental pollutants and found, after adjusting the model for temperature, squared temperature, and humidity, that emergency admissions for asthma increase by 78% with a three- to five-day lag following an increase in O_3_ concentration, especially during the hot season. Another study showed that an increase in O_3_ concentration due to the direct oxidation of cells, including inflammation and neural reflexes, reduces pulmonary function, increases the responses of the respiratory tract, and causes chronic obstructive pulmonary diseases (COPD), which can themselves be one of the main reasons for the prevalence of asthma and bronchospasm attacks during periods of rise in O_3_ concentration [32]. In another study [33], Wang et al. (1999) showed a relationship between increased O_3_ concentrations and the increased prevalence of asthma in men.

Ozone (O_3_) per se is a short-lived greenhouse gas and its predictable values increase with climatic changes. This pollutant is a highly potent oxidant created by the effect of sunlight on nitrogen dioxide and the production of atomic oxygen radical in the air. The maximum 24-hour concentration of this type of ozone (called tropospheric ozone) naturally reaches 0.06 ppm [34]. Ozone enters the body mainly through breathing and no other ways have been detected for its entry into the human body. Ozone can penetrate any part of the lung tissue depending on its initial concentration. The highest dose of ozone penetrates this tissue in the area between the bronchioles and the alveoli, and only a very small amount of ozone actually enters the blood; therefore, a small increase in the amount of ozone penetrating the body has a small effect on the trachea and bronchi, but a tangible effect on the main part of the lung [35].

In assessing the relationship between air pollutant concentrations and emergency hospital visits for asthma and bronchospasm, the adjusted multivariate models showed that each unit of increase in NO concentration led to a significant increase in the number of emergency visits during the epidemic periods. Each unit of increase in NO_2_ concentration also led to a significant increase in the number of emergency visits for asthma and bronchospasm attacks during the nonepidemic periods. As for the role of NO_2_, many studies have revealed a relationship between NO_2_ and the prevalence of asthma [36–38]. A study conducted in the US showed a 35% increase in the likelihood of wheezing and a 47% increase in the likelihood of shortness of breath with a one-hour 50-ppb increase in nitrogen [39]. In one study [27], Ho et al. (2007) showed that NO contributes to asthma attacks [27]. In a study conducted in Toronto, Canada [40], Jerrett et al. (2009) by a model which was adjusted for age, gender, Body Mass Index, and predicted percentage of Forced Vital Capacity showed that for every IQR increase in the mean concentration of NO_2_ (IQR is about 1 *µ*g/m^3^), relative risk (RR) for death due to respiratory diseases is 1.1 (0.83–1.46). Evidence suggests that 15 to 30 minutes of exposure to 500 *µ*g/m^3^ of NO_2_ activates asthmatic reactions [41]. Some studies have shown that 15 minutes of exposure to 500 *µ*g/m^3^ of NO_2_ in congested areas can cause inflammatory allergic reactions in the airways asymptomatically or without pulmonary defects [42].

Of the seven nitrogen oxides existing, NO and NO_2_ are the most important ones contributing to air pollution and affecting human health. NO and NO_2_ are collectively known as NO_*x*_ due to their ability to transform in photochemical smog reactions [35]. These pollutants disperse into the atmosphere from motor vehicles, power plants, burning natural gases, oil burners, atmospheric electrical discharge, biological processes involving bacteria, and factories producing chemicals such as nitric acid [43]. NO has respiratory effects such as impaired olfactory function, lethargy, fatigue, nasal irritation, breathing problems, throat irritation, psychological problems, dilated pupils, increased cases of acute bronchitis, and nitrosamine synthesis [43]. In its atmospheric concentrations, NO_2_ is only potentially stimulating and also potentially associated with COPD. It is also associated with the increased reaction of the airways in people with asthma, and acute respiratory symptoms such as coughing, chest pain, shortness of breath, and bronchopneumonia [44]. NO_2_ penetrates the entire respiratory system; however, studies have shown the main site for its reactions to be the end bronchioles. The main mechanism by which NO_2_ affects the respiratory system is involvement in lipid peroxidation in the cell membrane [45]. NO_2_ also has a boosting effect on the asthmatic response to exposure to allergens [11].

The results also showed a significant relationship between SO_2_ concentration and emergency hospital visits for asthma and bronchospasm attacks during the epidemic periods.

In a study conducted in six European cities [46], Andersen et al. (1997) showed that an increase of 50 *µ*g/m^3^ in the mean daily concentration of SO_2_ and particulates was associated with an increase in the daily frequency of hospital admissions for COPD. Other studies, including those by Wilson et al. in 2005 [29], Sheppard et al. in 1980 [47], and Linn et al. in 1987 [48], also showed that increased concentrations of SO_2_ cause bronchoconstriction and are associated with acute obstructive attack in asthmatic patients and an increased number of emergency hospital visits.

SO_2_ appears to have a major role in increasing hospital admissions and exacerbating COPD [49]. More than 80% of SO_2_ enters the atmosphere through the burning of fossil fuels by humans. SO_2_ is estimated to remain in the air for an average of two to four days and its general effects include airway constriction, bronchospasm, irritation of the eyes and the respiratory tracts, reduced respiratory efficiency and shortness of breath, reduced depth of breathing, reduced pulmonary defense, and ultimately the exacerbation of cardiovascular and respiratory complications [50, 51]. An SO_2_ concentration of 1 to 5 ppm causes obvious symptoms such as shortness of breath in some asthmatic patients within ten minutes and requires bronchodilation; at concentrations of 0.5 to 1 ppm, the individual experiences wheezing and difficulty in breathing within ten minutes of exposure [52, 53].

The present study also showed that, per each unit of increase in the mean daily relative humidity and evaporation, the risk of emergency hospital visits for bronchospasm increases during epidemic periods; however, each unit of increase in the mean daily temperature and wind speed reduces these visits by 84% and 54% during the same period. In one study [54], Kanaya (2001) showed a remarkable relationship between asthma and climatic and pollutant conditions. Lam et al. (2016) also argued that increased asthma is directly related to high humidity and cold weather [30]. Whittemore and Korn (1980) argued that, in cold weather, air pollutants increase the prevalence of asthma and bronchospasm attacks [55]. Tromp (1968) then argued that hospital visits by asthmatic people increase in humid weather [56]. In another study, Gonzalez-Barcala et al. (2013) showed that low humidity and high temperature have an inverse relationship with the rate of hospitalization in asthmatic patients [57]. This finding confirms the results of the present study, in which warmer weather (observed in the nonepidemic months) was associated with a lower frequency of hospital visits for asthma and bronchospasm attacks; it can therefore be assumed that respiratory allergies and asthma and bronchospasm attacks increase with the reduction in temperature accompanied with high humidity and no wind.

These results can be explained by the fact that all the examined pollutants can cause asthma and bronchospasm or affect their recurrence both independently and following precipitation; however, the present study found no relationships between pollutant concentrations (except for O_3_) and rainfall or thunderstorm-associated asthma and bronchospasm; other factors may be at play in causing these epidemics with the early autumn rainfall in Ahvaz aside from these pollutants—at least from most of them.

It can thus be argued that although air pollutants are associated with the prevalence and recurrence of asthma or bronchospasm, they are not considered the main cause of the thunderstorm-associated asthma or bronchospasm epidemics that have occurred in Ahvaz during the past five years; it may be that a combination of some of these pollutants and other factors such as plant allergens or their synergistic effects have caused these epidemics.

Pollutants such as SO_2_, CO, dust, smoke, organic volatile compounds, and heavy metals have been shown to play a role in increasing symptoms of allergy in sensitive people and play an important role as adjuvant in increasing allergenicity; the pollen given off by plants during the pollen season, which is one of the main causes of allergic rhinitis, allergic asthma, and allergic dermatitis, is more widespread in areas with industrial and vehicular pollutants [58–60]. There is strong evidence suggesting that asthma and allergic diseases increase significantly with the first thunderstorms occurring in the pollen season, and people with allergies are overexposed to these pollens during this period and therefore experience asthma or bronchospasm attacks [5, 23, 28, 61–67].

The Ahvaz metropolis has long been exposed to the pollutants created by the industrial manufacturing of steel, sugar cane, gas, and oil. According to the reports of the Environmental Protection Agency, the pollination of plants and weeds in Khuzestan and Ahvaz peaks from late September to late November every year; however, the actual amount of plant pollens in the air at different times of the year, especially during the epidemic and nonepidemic periods of respiratory problems, has not yet been measured in Khuzestan and Ahvaz and is only now being considered.

Idani et al. suggested that it is possible that incompatible vegetation or the abundance of nonnative plants in an industrial environment is the main reason for the recent respiratory incidents in Khuzestan Province [18]. Perhaps, in industrial areas with more apparent air pollution, plant pollens can cause more respiratory emergencies than other environments. Meteorologically, the Khuzestan Province and particularly Ahvaz have prolonged hot months and a short period of cold from December to February. Therefore, the planting of some generation of plants which were resistant to hot weather was introduced a few years ago and, now, these new plants are the most abundant species in the green spaces of Ahvaz city.

In fact, it is hypothesized that synergistic effects of pollutants and pollination of these new generation of plants may cause more respiratory symptoms specially in susceptible patients.

One should not forget the newly emerging crisis of sandstorms in the region, which is also a crisis of the entire Middle East. These sandstorms appear to have been caused by changes in vegetation in surrounding regions, environmental changes due to industrial activities, and the poor regional coordination for stabilizing the sands flowing in from the surrounding deserts especially the noncooperation of neighboring countries such as Iraq, Kuwait, and Saudi Arabia.

Collectively, these reasons distinguish this particular geographical region of Iran from other major cities and the findings obtained are thus unique to Ahvaz.

The present case-control research may be the first attempt at studying one of the most polluted cities of the world, that is, Ahvaz [19]. Every existing air pollutant, including SO_2_, NO_*x*_, NO, O_3_, NO_2_, CO, and PM_10_, was investigated in this research. The limitations of the study include the inability to measure variables such as the amount of plant pollen in the two different periods due to the lack of access to a pollen measuring device.

## 5. Conclusion

Although this study shows that, in and by themselves, most air pollutants cannot cause the outbreak of rainfall-associated asthma or bronchospasm, considering the particular climatic conditions in Ahvaz, the increased pollination in this city, the many industrial pollutants in the region, and the sandstorms that increase pollutant concentrations beyond the permissible level in early autumn, reducing the problem of allergies in the short term requires the development of an alert system that warns people of rainfalls, thunderstorms, and lighting early in the autumn and urges them to stay at home on these days unless necessary, and to then wear masks if they need to go out. Preventive treatment protocols for reducing the disease burden should also be implemented for people with a history of allergy and asthma before the beginning of the epidemic period. Long-term plans include reducing the concentrations of air pollutants, especially industrial pollutants and microparticulates, and pruning plants before the pollination season.

## Figures and Tables

**Table 1:** A Comparison of the clinical and epidemiological characteristics of patients in the case and control group during 2011–2015.

Variables	Groups	Epidemic group patient's *n* (%)	Patients attending at other times *n* (%)	OR	95% CI	*P* value
Gender	Male	2445 (46.1%)	421 (46.0%)	1.004	0.873–1.156	0.951
	Female	2862 (53.9%)	495 (54.0%)			
Age	<20	398 (7.5%)	64 (7.0%)	1.079	0.821–1.419	0.585
	>20	4909 (92.5%)	852 (93.0%)			
Asthma history	Yes	2215 (41.7%)	447 (48.8%)	0.752	0.653–0.865	**<0.001** ^†^
	No	3092 (58.3%)	469 (51.2%)			
Allergic reaction history	Yes	667 (12.6%)	109 (11.9%)	1.064	0.858–1.321	0.572
	No	4640 (87.4%)	807 (88.1%)			
Drug history for asthma	Yes	2084 (39.3%)	374 (40.8%)	0.937	0.812–1.081	0.372
	No	3223 (60.7%)	542 (59.2%)			
Smoking	Yes	3422 (64.5%)	528 (57.6%)	1.334	1.157–1.539	**<0.001** ^†^
	No	1885 (35.5%)	388 (42.4%)			
Final decision	Hospitalization	487 (9.2%)	55 (6.0%)	1.582	1.186–2.110	**0.002** ^†^
	Discharge	4820 (90.8%)	861 (94.0%)			
Recurrence	Yes	4050 (76.3%)	684 (74.7%)	1.093	0.930–1.285	0.282
	No	1257 (23.7%)	232 (25.3%)			
Wheezing	Yes	1654 (31.2%)	263 (28.7%)	1.124	0.963–1.312	0.137
	No	3653 (68.8%)	653 (71.3%)			
Dyspnea	Yes	5209 (98.2%)	900 (98.3%)	0.945	0.554–1.611	0.835
	No^†^	98 (1.8%)	16 (1.7%)			
Cough	Yes	3297 (62.1%)	583 (63.6%)	0.937	0.810–1.084	0.380
	No	2010 (37.9%)	333 (36.4%)			
Sputum	Yes	861 (16.2%)	201 (21.9%)	0.689	0.580–.819	**<0.001** ^†^
	No	4446 (83.8%)	715 (78.1%)			

^†^Maybe cough variant asthma.

**Table 2:** Comparing the levels of air pollutants and climate factors during the epidemic and nonepidemic periods in Ahvaz from 2011 to 2015.

Variable	Period	Mean	SD	*t*	*P* value
O_3_ (ppm)	Epidemic	32.4356	26.5714	3.454	**0.001**
	Nonepidemic	26.5714	8.67588		
PM_10_ (*µ* g/m^3^)	Epidemic	170.660	250.3346	−2.132	**0.034**
	Nonepidemic	228.906	384.5480		
NO (ppm)	Epidemic	29.2710	22.67823	−4.836	**<0.001**
	Nonepidemic	44.1286	46.19925		
NO_2_ (ppm)	Epidemic	27.7016	17.01837	−6.500	**<0.001**
	Nonepidemic	39.3536	24.76065		
NO_*x*_ (ppm)	Epidemic	53.2486	37.36753	−6.141	**<0.001**
	Nonepidemic	80.4071	63.98548		
CO (ppm)	Epidemic	1025.5676	839.06828	1.539	0.124
	Nonepidemic	930.6324	604.64199		
SO_2_ (ppm)	Epidemic	17.7463	8.34030	1.839	0.066
	Nonepidemic	16.2249	11.10837		
Temperature (°C)	Epidemic	19.250	3.8260	1.655	0.055
	Nonepidemic	17.200	2.6142		
Relative humidity (%)	Epidemic	58.86	9.591	0.884	**0.041**
	Nonepidemic	55.86	8.328		
Total rainfall (mm)	Epidemic	35.329	34.2287	1.086	**<0.001**
	Nonepidemic	23.407	22.7236		
Total evaporation (mm)	Epidemic	104.429	40.8523	−1.020	**<0.001**
	Nonepidemic	119.286	36.0956		
Wind speed (m/s)	Epidemic	12.50	9.452	−0.345	0.102
	Nonepidemic	13.57	6.745		

**Table 3:** Results of crude and adjusted negative binomial regression and of the effect of pollutants and climate factors on respiratory hospital admissions (ratio of increase in hospital admissions in day per unit of increase in pollutants and climate factors daily average) in the epidemic periods.

Variables	Crude IRR and 95% CI	*P*	Adjusted IRR and 95% CI	*P*
O_3_ (ppm)	1.000 (0.995–1.005)	0.982	1.002 (0.998–1.007)	0.345
PM_10_ (*µ* g/m^3^)	1.000 (0.999–1.000)	0.293	1.000 (0.999–1.001)	0.506
NO (ppm)	**1.007 (1.001**–**1.012)**	**0.013**	**1.008 (1.001**–**1.016)**	**0.037**
NO_2_ (ppm)	0.996 (0.989–1.002)	0.201	0.993 (0.983–1.003)	0.172
NO_*x*_ (ppm)	1.002 (0.999–1.006)	0.191	1.001 (0.997–1.006)	0.503
CO (ppm)	1.000 (1.000–1.000)	0.424	1.000 (1.000–1.000)	0.618
SO_2_ (ppm)	1.009 (0.999–1.020)	0.085	**1.014 (1.000**–**1.028)**	**0.044**
Temperature (°C)	0.935 (0.867–1.008)	0.078	**0.163 (0.076**–**0.351)**	**<0.001**
Relative humidity (%)	1.029 (0.995–1.064)	0.095	**1.418 (1.041**–**1.932)**	**0.027**
Total rainfall (mm)	**1.056 (1.012**–**1.103)**	**0.012**	1.154 (0.963–1.384)	0.120
Total evaporation (mm)	0.998 (0.994–1.001)	0.195	**1.130 (1.072**–**1.191)**	**<0.001**
Wind speed (m/s)	1.100 (0.918–1.318)	0.303	**0.461 (0.233**–**0.915)**	**0.027**

**Table 4:** Results of crude and adjusted negative binomial regression and of the effect of pollutants and climate factors on respiratory hospital admissions (ratio of increase in hospital admissions in day per unit of increase in pollutants and climate factors daily average) in the nonepidemic periods.

Variables	Crude IRR and 95% CI	*P*	Adjusted IRR and 95% CI	*P*
O_3_ (ppm)	**1.018 (1.001**–**1.034)**	**0.036**	1.012 (0.992–1.032)	0.241
PM_10_ (*µ*g/m^3^)	1.000 (1.000–1.000)	0.692	1.000 (1.000–1.000)	0.684
NO (ppm)	0.999 (0.996–1.002)	0.613	0.998 (0.994–1.003)	0.477
NO_2_ (ppm)	1.005 (0.999–1.010)	0.100	**1.010 (1.001**–**1.019)**	**0.023**
NO_*x*_ (ppm)	1.000 (0.998–1.002)	0.727	1.002 (0.999–1.005)	0.157
CO (ppm)	1.000 (1.000–1.000)	0.646	1.000 (0.999–1.000)	0.067
SO_2_ (ppm)	0.990 (0.978–1.002)	0.101	0.988 (0.972–1.004)	0.153
Temperature (°C)	1.041 (0.953–1.137)	0.374	0.867 (0.186–4.038)	0.855
Relative humidity (%)	0.979 (0.944–1.015)	0.254	1.318 (0.786–2.210)	0.296
Total rainfall (mm)	0.974 (0.927–1.023)	0.289	0.685 (0.392–1.198)	0.185
Total evaporation (mm)	1.002 (0.998–1.006)	0.287	1.008 (0.939–1.083)	0.819
Wind speed (m/s)	1.100 (0.677–1.786)	0.701	2.775 (0.549–14.034)	0.217
